# Role of the Gut in Diabetic Dyslipidemia

**DOI:** 10.3389/fendo.2020.00116

**Published:** 2020-03-13

**Authors:** Priska Stahel, Changting Xiao, Avital Nahmias, Gary F. Lewis

**Affiliations:** Departments of Medicine and Physiology and Banting & Best Diabetes Centre, University of Toronto, Toronto, ON, Canada

**Keywords:** intestine, chylomicron, Enteroendocrine, lipoprotein, diabetes

## Abstract

Type 2 diabetes (T2D) is associated with increased risk of cardiovascular disease (CVD). In insulin resistant states such as the metabolic syndrome, overproduction and impaired clearance of liver-derived very-low-density lipoproteins and gut-derived chylomicrons (CMs) contribute to hypertriglyceridemia and elevated atherogenic remnant lipoproteins. Although ingested fat is the major stimulus of CM secretion, intestinal lipid handling and ultimately CM secretory rate is determined by numerous additional regulatory inputs including nutrients, hormones and neural signals that fine tune CM secretion during fasted and fed states. Insulin resistance and T2D represent perturbed metabolic states in which intestinal sensitivity to key regulatory hormones such as insulin, leptin and glucagon-like peptide-1 (GLP-1) may be altered, contributing to increased CM secretion. In this review, we describe the evidence from human and animal models demonstrating increased CM secretion in insulin resistance and T2D and discuss the molecular mechanisms underlying these effects. Several novel compounds are in various stages of preclinical and clinical investigation to modulate intestinal CM synthesis and secretion. Their efficacy, safety and therapeutic utility are discussed. Similarly, the effects of currently approved lipid modulating therapies such as statins, ezetimibe, fibrates, and PCSK9 inhibitors on intestinal CM production are discussed. The intricacies of intestinal CM production are an active area of research that may yield novel therapies to prevent atherosclerotic CVD in insulin resistance and T2D.

## Introduction

Atherogenic dyslipidemia is characterized by hypertriglyceridemia, elevated small, dense LDL particles, reduced HDL, elevated remnant lipoproteins and postprandial hyperlipidemia (1). This complex of lipid abnormalities is associated with underlying insulin resistance (IR) and has an increased prevalence in type 2 diabetes (T2D). The exaggerated postprandial lipemia in individuals with T2D or IR is attributed to elevated liver- and intestine- derived triglyceride-rich lipoproteins (TRL) due to decreased lipoprotein clearance and/or increased secretion. Intestinally-derived chylomicron (CM) particles can be quantified by the presence of an apolipoprotein B48 (apoB48) singularly present on each particle. ApoB48 is detected in atherosclerotic plaque demonstrating that CM remnants can penetrate the endothelium and contribute to lesion formation (2).

Dysregulation of TRL clearance from the circulation has been extensively reviewed elsewhere (3) and will not be the focus of this review. However, it is important to note that postprandial accumulation of lipoproteins and lipoprotein remnants is largely influenced by clearance capacity (4). CMs and liver-derived very-low-density lipoproteins (VLDL) compete for delipidation by lipoprotein lipase (LPL) and for subsequent saturable hepatic remnant removal (5, 6). In IR, lipoprotein clearance is diminished, in part due to decreased LPL activity, altered lipoprotein composition, reduced hepatic clearance and remnant removal or increased TRL pool size resulting in more competition for clearance (7). In addition, insulin mediated activation of LDLR-related protein-1 which is involved in CM remnant clearance is blunted in insulin-resistant mice (8). Although clearance is an important factor to consider in diabetic dyslipidemia, this review will focus on our current understanding of CM production by the gut in IR states and T2D. We will discuss how existing therapeutic strategies targeting dyslipidemia influence CM secretion and the potential utility of novel therapeutics to specifically reduce CM production. We acknowledge that few studies have examined intestinal lipoprotein secretion in diabetic animal models and humans; therefore at the present time we extrapolate from healthy and insulin resistant animal models and humans, with the understanding that our knowledge of intestinal lipoprotein secretion in diabetes is far from complete and will undoubtedly require revision as our knowledge expands.

## Physiological Intestinal Lipid Handling and CM Secretion

To understand the role of the intestine in diabetic dyslipidemia, we will briefly review normal lipid processing by the gut, which has been extensively reviewed elsewhere (9, 10). Dietary triglycerides (TGs) are hydrolyzed to monoacylglycerol (MAG) and fatty acids (FAs) in the intestinal lumen. Several putative FA transporters have been identified including CD36/FAT. FA binding proteins (FABP) such as I-FABP and L-FABP are involved in intracellular FA transport (9).

Re-esterification occurs primarily by the monoacylglycerol pathway through sequential esterification by monoacylglycerol acyltransferase (MGAT) and diacylglycerol acyltransferase (DGAT) between the leaflets of the endoplasmic reticulum (ER) membrane (11). This pathway contributes the majority of TG for CM synthesis while *de novo* lipogenesis (DNL) and the glycerol phosphate pathway are additional contributors. TGs enter the ER lumen to fuse with lipid-poor apoB48-containing particles to form prechylomicrons, which also contain cholesteryl esters and acquire apolipoprotein AIV (apoAIV) (12). This lipidation process is mediated by microsomal triglyceride transfer protein (MTP). Prechylomicrons are transported to the Golgi in prechylomicron transport vesicles with fusion occurring at the Golgi facilitated by various SNARE proteins. Mature CMs are subsequently exocytosed at the basolateral membrane, each containing a single apoB48 along with apoAI and apoAIV. During active lipid absorption the basement membrane underlying enterocytes may become leaky to facilitate movement of CMs into the lamina propria (13, 14). Large porous junctions in lacteals allow CMs to enter the lymphatic vasculature for eventual delivery to the circulation. Rather than serving as a passive conduit, lacteals and lymphatic ducts are gaining attention as active regulatory sites in CM transport (15). As CMs circulate, interactions with other lipoproteins facilitate the exchange of apolipoproteins allowing CM particles to acquire apoE, apoC2, and apoC3, which regulate hepatic CM removal and delipidation by LPL (16).

Although the absorption of dietary TG is highly efficient, not all TGs are immediately released from enterocytes. Following re-esterification at the ER, a portion of TGs bud off the ER membrane into the cytosolic space to form cytosolic lipid droplets (CLDs), which serve as transient lipid storage pools within enterocytes. CLDs and other enteral lipid stores are evident in human intestinal tissue for many hours after a meal and can be mobilized by a number of stimuli (17–19). Several factors including dietary macronutrient load, circulating FA, glucose, insulin, and gut hormones glucagon-like peptide-1 (GLP-1) and glucagon-like peptide-2 (GLP-2) have been identified as key players in the complex fine tuning of CM appearance in the early prandial phase as well as in mediating CLD mobilization several hours after a meal (9, 15). Interestingly, the co-secreted gut hormones GLP-1 and GLP-2 have opposing effects on CM secretion, with GLP-1 inhibiting and GLP-2 promoting appearance of apoB48 lipoproteins. Enterocytes do not express receptors to either GLP-1 or GLP-2, therefore their modulation of CM secretion is through indirect mechanisms that remain to be fully elucidated (20).

## Increased CM Production in Prediabetic, Insulin Resistant States

Prevalence of unfavorable postprandial hypertriglyceridemia (postprandial TG > 220 mg/dl) increases progressively from non-diabetic to prediabetic to T2D states in humans and was linked to increasing severity of hepatic IR (21). Both increased CM production and decreased clearance have been observed in insulin resistant states (1). We previously demonstrated increased production rate of apoB48-containing TRLs in insulin resistant humans (22, 23). Couture et al. demonstrated a 102% increase in TRL-apoB48 pool size and 87% increase in production rate in IR compared to insulin sensitive obese men in the fed state (23). A trend toward decreased apoB48 clearance rate was observed but did not reach statistical significance, while VLDL apoB-100 clearance was significantly reduced (23). When quantifying TG rather than apoB48 kinetics, men with metabolic syndrome had elevated fed-state VLDL-TG and CM-TG due to increased production rates (24). Decreased CM clearance has also been observed in conditions of obesity and T2D, contributing to the elevated TRL concentrations in these states (25–27). In contrast to the increased secretion of CM that characterizes IR, which is usually also accompanied by chronic hyperinsulinemia, in the experimental hyperinsulinemic-euglycemic clamp setting, acute hyperinsulinemia suppresses CM secretion in healthy humans directly and in part by suppressing circulating FA (28). In animal models, intestinal IR can be elicited by high-fat (29) or high-fructose feeding (30, 31). A single oral bolus of palmitate in mice is sufficient to impair insulin suppression of CM production, possibly via ceramide-mediated inhibition of Akt signaling (32). This is consistent with ceramide inhibition of insulin signaling in muscle, liver and adipose. Therefore, despite the rapid turnover of enterocytes *in vivo*, persistent overnutrition including increased consumption of saturated FAs, may induce intestinal IR, with one possible mechanism being increased ceramide production. Indeed, plasma and tissue ceramide are elevated in humans in IR and T2D and upon excess saturated fat intake (33). From animal models it is apparent that intestinal IR increases CM synthesis with increased expression and activity of key proteins involved in lipogenesis and CM secretion, namely MTP, MGAT, DGAT, apoAIV, and sar 1 GTP ase (30, 34). The consequences of impaired intestinal insulin sensitivity are increased DNL, excess CM production and secretion (31, 35).

In accordance with animal models, humans with severe IR or diabetes have elevated intestinal MTP expression and protein abundance compared to more insulin sensitive individuals (36, 37). Individuals undergoing bariatric surgery who are markedly insulin resistant (HOMA-IR > 7) had decreased duodenal insulin signaling capacity as indicated by decreased phosphoAKT abundance and higher p38 MAPK compared to obese, more insulin sensitive patients (HOMA-IR < 3) (36). The insulin resistance was postulated to be caused by greater oxidative stress and inflammation, increased markers of which were observed in the diabetic duodenum. This perturbed metabolic state may be causal to the increased DNL and apoB48 biogenesis in IR (36). In contrast, when assessed in humans with modest IR, key intestinal genes involved in FA transport, and lipid/lipoprotein metabolism including SREBP-2, MTP, and DGAT2 were downregulated (23). In particular, the observed 25% decrease in MTP protein is intriguing in light of increased CM production rate in the same subjects. To address this discrepancy, the authors postulated that in modest IR states hyperinsulinemia may suppress MTP expression via the insulin responsive element of the MTP promoter region. In contrast, relative insulin insufficiency in T2D may allow for greater MTP expression (23). We speculate that in modest IR with normoglycemia total efficacy of insulin modulation of lipid metabolism may be fully intact whereas more severe IR insulin modulation of lipid metabolism may be significantly impaired leading to altered lipid metabolism. In both states, modest and severe IR, adipose IR increases circulating FA which may serve as substrates for intestinal CM synthesis and thus adaptive suppression of enterocyte DNL and fat absorption may occur (23, 38). Indeed, men with metabolic syndrome had a greater contribution to CM-TG from non-oral FA than lean controls (24). Sources of non-oral FA may include circulating FA, pre-existing TG stores (e.g., enterocyte CLDs), or TG arising from DNL (24).

In the postprandial state, enteroendocrine L-cells lining the intestine secrete GLP-1 and GLP-2 in equimolar amounts in response to nutrients (39). GLP-1 has diverse physiological roles, including potentiation of glucose-stimulated insulin secretion, appetite suppression and inhibition of gastric emptying (40). In contrast, GLP-2 acts as an intestinotrophic hormone, stimulating intestinal proliferation and aiding in intestinal repair processes. Despite equimolar secretion, GLP-1 and GLP-2 have opposing effects on CM secretion. In animal models, intravenous GLP-1 reduced postprandial apoB48 and TG concentrations (41). In Phase 2/3 clinical assessments, GLP-1 analogs lower CM production in healthy humans whereas (42) pharmacological doses of GLP-2 robustly stimulate CM secretion in human and animal models (43–46). In normal, insulin sensitive states and in fructose-fed hamsters the effects of GLP-2 predominate with elevated postprandial lipemia (41).

High-fat feeding to induce IR in mice also induces leptin resistance as evidenced by a loss of the inhibitory effect of leptin on food intake (47). Leptin resistant animals have decreased glucose-stimulated GLP-1 secretion and fasted GLP-1 (47), suggesting leptin resistance may contribute to impaired GLP-1 secretion in obese humans. GLP-1 reduces postprandial apoB48 and TG (41, 42), therefore impaired GLP-1 secretion due to leptin resistance could be a contributing factor to elevated CM secretion. Leptin is classically described as an important satiety signal produced by the expanding adipocyte in the setting of net positive energy balance. Leptin is also secreted to a lesser extent from the gastric mucosa into the gastric lumen and circulation. Gastric leptin partially escapes hydrolysis and enters the intestinal lumen to bind the leptin receptor expressed on the luminal side of intestinal epithelial cells (48). Jejunally infused leptin has been shown to regulate intestinal MTP in the mouse independently of vagal innervation (49). In this regard, gastric leptin secretion in the early prandial phase may be important for regulating CM production and leptin resistance may be another contributing factor to increased CM production in IR states.

## Dysregulation of Intestinal Lipoprotein Metabolism in Type 2 Diabetes

IR is a prominent feature of T2D, accompanied by relative or absolute pancreatic insulin secretory insufficiency resulting in hyperglycemia, with plasma insulin concentrations ranging from elevated to low. Much of the above discussion of abnormal intestinal lipoprotein metabolism in IR therefore also pertains to those with T2D. In fact in patients with T2D, the acute inhibitory effect of insulin on apoB48 production is blunted (50). Similarly, the stimulatory effect of insulin on intestinal glucose uptake in obese subjects is diminished, although this was improved by bariatric surgery (51). In obese subjects with T2D, fasting and postprandial TGs were significantly reduced 2 weeks after bariatric surgery. Most importantly, the incremental area under the curve of postprandial plasma TGs decreased by 60% compared to pre-surgery (52). In a detailed study involving kinetic assessment in the constant fed state in obese, non-diabetic humans, TRL-apoB48 concentration was significantly reduced, with a reduction in TRL-apoB48 when assessed 6 months post-sleeve gastrectomy compared to before surgery (53). While reduced dietary intake likely contributes to reduced TG post-surgery, this may also be partially mediated by improved intestinal function perhaps by restored intestinal insulin sensitivity. In addition, hyperchylomicronemia in diabetic dyslipidemia may be further exasperated by blunted insulin suppression of hepatic apoB100 production (50, 54), increasing competition for clearance and thereby increasing plasma retention time.

Hyperglycemia itself may further enhance CM secretion in the diabetic state. In healthy, non-diabetic adults, we have shown enhanced apoB48 production in response to intraduodenal and intravenous glucose, and in response to intraduodenal fructose (55, 56). Whether hyperglycemia in diabetes contributes directly to enhanced CM secretion is unknown.

In addition to competition for clearance, altered CM apolipoprotein composition in diabetes may also contribute to delayed clearance and increased atherosclerotic risk in T2D. In animal models, CMs originating from diabetic animals are cleared more slowly than those from non-diabetic animals, possibly due to reduced apoE abundance on the CM particle (57). ApoAIV is produced primarily in the intestine in response to an oral lipid load and is increased in the circulation in IR (58). Increased plasma apoAIV has been observed association with hypertriglyceridemia in patients with T2D (59). In the circulation a portion of apoAIV associates with HDL or circulates lipid-free to influence glucose and lipid metabolism. Glycation of apoAIV in circulation is associated with coronary artery disease severity in patients with T2D (60). The extent to which alterations in apoAIV production by the intestine influence postprandial metabolism in T2D remains to be fully elucidated. ApoAIV knockout mice secrete larger CMs into the lymph (12) and thus altered apoAIV production may influence the rate of delipidation through alterations in CM size as larger CMs are lipolyzed at a faster rate than smaller particles in animal models (61, 62). The multitude of metabolic functions of apoAIV are an active area of research that may improve our understanding of the regulation of intestinal apoAIV production and its influence on lipoprotein clearance in metabolic disease (63).

## Effects of Currently Approved Lipid Modifying and Antidiabetic Therapies on Gut Lipid Handling in Insulin Resistant States and Diabetes

**Figure 1:**
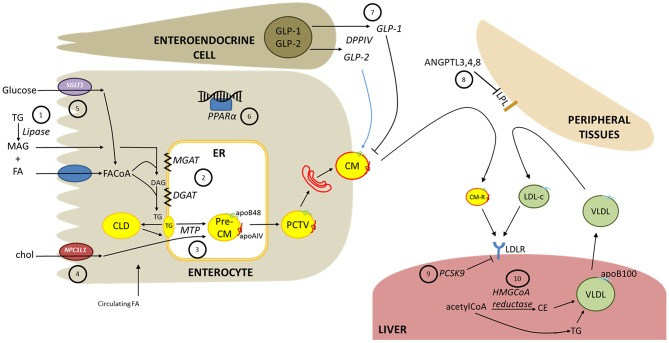
Intestinal lipoprotein overproduction in diabetes and potential therapeutic targets. The following are potential therapeutic targets, as discussed in the text. **(1)** Dietary triglycerides (TG) are hydrolyzed in the intestinal lumen by pancreatic lipases which can be inhibited to reduce fatty acid (FA) and monoacylglycerol (MAG) availability for absorption. **(2)** Fatty acyl-CoA (FACoA) are progressively re-esterified into TG by monoacylglycerol acyltransferase (MGAT) and diacylglycerol acyltransferase (DGAT) **(3)** Microsomal triglyceride transfer protein (MTP) is a crucial enzyme for lipidation of nascent apoB48 and the pre-chylomicron (CM) particle **(4)** The cholesterol transporter Niemann-Pick C1-like-1 (NPC1L1) is a rate limiting step in cholesterol absorption. Cholesteryl esters are an important component of CM particles. **(5)** Antagonism of sodium-glucose cotransporter-1 (SGLT1) is in development as a diabetes therapy to attenuate glucose absorption which may modify CM secretion. **(6)** Fibrates are approved lipid-lowering treatments that agonize peroxisome proliferator-activated alpha (PPARα). Whether fibrates markedly lower CM secretion is under investigation. **(7)** Glucagon-like peptide-1 (GLP-1) suppresses CM secretion. GLP-1 analogs and inhibitors of the GLP-1-degrading enzyme DPP-IV are available diabetes therapies that acutely inhibit CM secretion in healthy humans. Glucagon-like peptide-2 (GLP-2) has been shown to acutely stimulate intestinal CM release in human and rodent models, however antagonistic therapies have not yet been examined as a therapy to decrease CM secretion. **(8)** Circulating CMs and liver-derived very-low density lipoproteins (VLDL) are delipidated by lipoprotein lipase (LPL) at peripheral tissues. Angiopoietin-like peptides (ANGPTL) 3,4 and 8 negatively regulate LPL and their effects on CMs is under investigation. **(9)** Hepatic clearance of CM remnants (CM-R) and low-density lipoprotein cholesterol (LDL-c) is mediated in part by the LDL receptor (LDLR). Proprotein convertase subtilisin/kexin type 9 (PSCK9) negatively regulates LDLR and is also highly expressed in the small intestine. Monoclonal antibodies against PCSK9 effectively lower LDL-c; however, PCSK9 inhibition in healthy humans had no impact on postprandial TG or apoB48 kinetics. **(10)** Statins are well-described inhibitors of HMGCoA reductase that effectively reduce cholesterol synthesis. Statin therapy reduces circulating apoB48 and CM-TG by increasing CM clearance, and perhaps decreasing CM secretion.

Statin treatment is effective in primary (64) and secondary (65) CV event prevention in patients with T2D. The effects of statin treatment on CV events reduction show a linear relationship between CV events reduction and LDL-c reduction suggesting that a possible effect of statin on CM secretion is likely to be a minor factor of the anti-atherosclerotic benefits of the statins. However, it is interesting to note that statins also have minor effects in modulating intestinal lipoproteins (Figure 1, #10). Four weeks of cerivastatin therapy significantly decreased postprandial CM apoB48 in patients with T2D (66). Similarly, 6 weeks of atorvastatin treatment in obese men significantly reduced circulating apoB48 and remnant-like particle cholesterol following remnant-like emulsion infusion, perhaps due to increased hepatic clearance (67). Atorvastatin treatment in healthy, normolipidemic men decreased postprandial apoB48 by increasing clearance and decreasing secretion after a fat load (68). Statin-mediated inhibition of cholesterol synthesis induced compensatory increases in cholesterol absorption by increasing intestinal expression of Niemann-Pick C1-like 1 protein (NPC1L1) (69). Therefore, a common add-on therapy to statin is ezetimibe which inhibits NPC1L1 (Figure 1, #4). In patients with T2D, compared to simvastatin alone, adding ezetimibe produced significantly fewer CMs that were cholesterol-poor and decreased fasting and postprandial CM-TG (70). Similarly, when patients with T2D failed to reach LDL-c targets on simvastatin alone, addition of ezetimibe improved lipoprotein profile (71). Over and above the well-documented effects of statins in upregulating LDL-receptor-mediated clearance of LDL particles from the circulation, statin therapy may also exert some of its benefits through improved metabolism of postprandial lipoproteins including apoB48 CM remnants.

Fibrates are an alternative lipid-lowering option for the treatment of dyslipidemia; however, their questionable cardiovascular benefit limits their widespread use (72, 73). The transcription factor PPARα is highly expressed in the intestine and is activated by several natural ligands, particularly polyunsaturated FAs (74) (Figure 1, #6). It regulates the expression of several apolipoproteins and genes involved in FA metabolism including the transcription factor sterol regulatory element binding protein-1c (SREBP-1c) (74). Recently, a more potent and selective PPARα agonist called pemafibrate (K-877) has been developed that lowers plasma TG to a similar extent as fenofibrate but with minimal adverse events (75, 76). In a phase III trial, 52 weeks of pemafibrate treatment reduced fasting TG by 45% compared to placebo in patients with T2D (77). Notably, pemafibrate significantly decreased apoB48 by up to 56% (77). This could be due to decreased CM production and/or increased clearance. In mice, pemafibrate decreased small intestine expression of apoB to a greater extent than fenofibrate while eliciting no change in hepatic apoB expression (78). In LDL receptor knockout mice, pemafibrate decreased apoC3 expression and increased plasma LPL activity suggesting increased clearance capacity (79). Similarly, Araki et al. observed decreased plasma apoC3 in patients with T2D treated with pemafibrate (77). These studies suggest that pemafibrate could effectively modulate CM production to treat diabetic dyslipidemia. The ongoing Pemafibrate to Reduce Cardiovascular Outcomes by Reducing Triglycerides in Patients with Diabetes (PROMINENT) study will evaluate major adverse cardiovascular events in T2D with suboptimal LDL-c lowering by statins (NCT03071692) (80).

Proprotein convertase subtilisin/kexin type 9 (PCSK9) is highly expressed in the liver, kidney, and small intestine and negatively regulates cell surface receptors, decreasing LDLR in liver and intestine leading to blunted hepatic TRL clearance (81) (Figure 1, #9). PCSK9 inhibition by monoclonal antibodies is an effective therapeutic strategy shown to lower LDL-c. PCSK9 knockout animals show decreased intestinal apoB output with no difference in lymphatic TG output suggesting an increase in CM size (82). This effect may be mediated by increased MTP abundance (83). Kinetic studies in healthy humans have shown increased clearance and decreased production of LDL-apoB; however, there was no impact on postprandial TG or apoB48 kinetics with evolocumab or alirocumab treatment (68, 84).

A discussion about diabetes therapies would be incomplete without considering the effects of some glucose lowering, antidiabetic therapies on intestinal lipoprotein metabolism. Metformin, for example, accumulates in jejunal mucosa in humans and thus is likely to regulate metabolic processes of enterocytes (85). The observation that intravenous metformin does not improve glycemia to the same extent as oral metformin further supports a crucial role for the intestine as a site of metformin action (86). In patients with T2D poorly controlled by sulfonylureas, the addition of metformin significantly improved glycemic control and halved postprandial CMs and CM remnants (87). Metformin treatment in morbidly obese T2D patients decreased jejunal expression of SREBP-1c, acetyl-coA carboxylase involved in FA synthesis, and apoAIV involved in CM secretion, suggesting attenuation of DNL and lipoprotein synthesis by metformin (58). Metformin may indirectly attenuate CM secretion through increased production of GLP-1 or by delayed gastric emptying. Metformin increased fasting GLP-1 in patients with T2D (88, 89) potentially by modulating bile acid pools (88). Interestingly, timing of metformin delivery may alter TG responses as pre-meal metformin decreased postprandial TG to a greater extent than post-meal delivery in patients with T2D and increased satiety with no difference in GLP-1 (90).

GLP-1 receptor agonists are known to lower postprandial TG. A reduction in postprandial TRL-apoB48 has been demonstrated in humans with T2D and in animal models and may occur independently of plasma insulin and gastric emptying (91, 92) (Figure 1, #7). In healthy humans, TRL particle kinetics were evaluated in the fed state with continuous duodenal liquid meal infusion and a pancreatic clamp to avoid confounding effects of gastric emptying and the effects of other hormones such as insulin and glucagon. This revealed that a single dose of exenatide acutely decreased apoB48 production rate with no effect on catabolism (42). We have previously reviewed the effects of incretin therapies on intestinal lipoprotein metabolism (93). When CM-TG kinetics were assessed in patients with T2D, lixisenatide reduced CM-TG appearance after a single meal likely via delayed gastric emptying (94, 95). However, over the course of a 12-h feeding protocol lixisenatide reduced CM-TG through increased clearance with no effect on production rate (95). Following 6 months of liraglutide treatment in T2D, apoB48 production was significantly decreased and clearance was increased (96). Incubating jejunal explants from mice with liraglutide reduced expression of key CM synthesis genes including apoB48, DGAT1, and MTP (96). Similarly, exenatide suppressed apoB48 expression in hamster enterocytes suggesting direct effects of GLP-1 receptor signaling on CM synthesis (92). GLP-1 agonists improve CVD outcomes in T2D (97, 98), perhaps in part by modulating intestinal CM production.

Sodium-glucose co-transporter 2 (SGLT2) inhibitors, which enhance urinary glucose secretion, are approved therapeutics in T2D. Recently, cardiovascular outcome trials showed improvements in CV outcomes in response to dapaglifozin (99), canaglifozin (100) and empaglifozin (101) in patients with T2D. The underlying mechanisms of CV protection are unclear. Three month canaglifozin treatment in T2D did not alter LDL-c or CM-c but increased HDL-c (102). Canagliflozin treatment at clinical doses modestly and transiently inhibits intestinal SGLT1, an important glucose transporter on the luminal side of enterocytes (103, 104) (Figure 1, #5). In humans, canagliflozin increased postprandial GLP-1 and peptide YY (PYY) (104), perhaps by increasing glucose delivery to the distal gut which has a higher density of incretin producing cells. In accordance with this, acarbose treatment which inhibits alpha glucosidase increases carbohydrate delivery to the distal gut and increases GLP-1 secretion (105). Similarly, specific SGLT1 inhibition in humans delayed intestinal glucose absorption and reduced GIP secretion while increasing GLP-1 and PYY secretion (106). Theoretically, SGLT1 inhibition could blunt early post-meal intestinal CM secretion by decreased glucose availability for DNL or by increased GLP-1-mediated inhibition of CM secretion, however gut DNL is likely a minor contributor to CM-TG. Dual inhibitor compounds targeting both SGLT2 and SGLT1 are under development, with pilot studies showing improved postprandial glucose control (107). Combining this dual antagonist with the DPPIV inhibitor sitagliptin further increased active GLP-1 and PYY in patients with T2D (108). Therefore, SGLT1 inhibition may serve as a novel therapeutic strategy in T2D with potential indirect benefits on CM secretion.

## Novel Therapies Targeting the Gut for the Treatment of Diabetic Dyslipidemia

Advances in our understanding of the intricacies of intestinal lipid handling and CM secretion as well as the atherogenic nature of CM remnant particles has led to the search for therapies to modify various aspects of CM production to ameliorate diabetic dyslipidemia. Beginning in the intestinal lumen, inhibition of gastric and pancreatic lipases by the drug orlistat (Figure 1, #1) developed for the treatment of obesity decreases intestinal TG absorption by 30% but adverse gastrointestinal (GI) effects were observed in 16–40% of patients (109). In T2D, orlistat significantly decreased TG and apoB compared to placebo (110). However, when given in combination with metformin improvements were observed in total cholesterol, LDL cholesterol and the LDL:HDL ratio but not in TG (111). Due to the relatively high incidence of adverse events and inconsistent efficacy, orlistat is not considered to be an effective therapy for treatment of diabetic dyslipidemia.

Inhibition of intestinal TG esterification is another avenue of active research to improve diabetic dyslipidemia (Figure 1, #2). Three isoforms of MGAT have been identified in humans and rodents. MGAT2 is highly expressed in the intestine of rodents and humans while MGAT3 is expressed only in the human intestine (112). Selective MGAT2 inhibition by orally administered small molecule inhibitor dose-dependently decreased postprandial CM-TG by up to 58% in mice (113). MGAT2 inhibition showed a similar phenotype to MGAT2 knockout and did not increase fecal fat suggesting no impact on fat absorption. Inhibition of TG re-esterification diverted FA toward beta oxidation in the small intestine (113). Human MGAT inhibitors have been identified by high-throughput screening methods (114), but it remains to be seen whether MGAT inhibition is efficacious and well-tolerated in humans and whether it has utility in treating diabetic dyslipidemia. Downstream of MGAT-mediated formation of diacylglycerol is DGAT, which catalyzes the final step in TG esterification. DGAT inhibition has been explored in human and animal models. Intestinal DGAT1 deficiency and pharmacological inhibition in mice decreased postprandial TG with delayed gastric emptying and inhibited CM secretion (115). Delayed gastric emptying may be attributable to increased GLP-1 secretion (116, 117). In humans, DGAT1 inhibition with pradigistat in patients with familial chylomicronemia syndrome decreased fasting TG primarily by decreasing CM-TG (118). However, adverse GI effects such as diarrhea were experienced by the majority of participants (118, 119). This is perhaps not surprising given that loss-of-function variants in DGAT1 are linked to congenital diarrheal disorder (120). In patients with T2D, pradigistat dose-dependently decreased total cholesterol, TG, LDL-c, and body weight (121). Other DGAT1 inhibitory compounds have been investigated. Single administration of the selective DGAT1 inhibitor PF-04620110 dose-dependently decreased postprandial TG in healthy humans (122). Grape extract contains components that decrease DGAT1 activity *in vitro* and was shown to reduce serum TG without GI side effects in overweight/obese but otherwise healthy humans after a high-fat meal (123). Structural changes to DGAT1 inhibitors or further development of DGAT1 antisense oligonucleotides may yield novel therapeutics that reduce postprandial TG without GI intolerability (124–126).

The MTP inhibitor lomitapide has been approved for a number of years for the treatment of homozygous familial hypercholesterolemia (HoFH) and has been used off label for the treatment of severe hypertriglyceridemia (127, 128). Lomitapide use is severely curtailed by side effects of diarrhea and liver fat accumulation (129). Intestine-specific MTP inhibition (Figure 1, #3) lowers CM secretion without affecting hepatic lipoprotein production and thereby minimizing hepatotoxicity and hepatosteatosis. In hamsters, the small molecule intestine-specific MTP inhibitor JTT-130 suppressed CM-TG following oil gavage without suppressing hepatic TG secretion. Importantly, repeated dosing for 2 weeks did not induce hepatotoxicity (130). However, hamsters treated with JTT-130 show impaired FA absorption with increased fecal FA and cholesterol content suggesting diarrhea may still be a complication of intestine specific MTP inhibition (131). In rats and apoE-/- mice, the small molecule inhibitor SLx-4090 specifically inhibited CM secretion with no effect on hepatic TG secretion and effectively reduced postprandial lipids (132, 133). Similarly in Caco-2 cells, SLx-4090 decreased apoB secretion (132). Preliminary reports of a phase 2 clinical trial (NCT00871936) SLx-4090 given to patients with T2D on metformin demonstrated 35% decreases in both postprandial TG and FA, compared to placebo (134). MTP inhibitors are not currently approved for use in T2D and for the foreseeable future will likely serve as orphan drugs for the treatment of HoFH or severe hypertriglyceridemia.

Novel therapeutics for atherogenic dyslipidemia are emerging; however, their effects on intestinal lipoprotein secretion require further investigation. Apolipoprotein C-III (apoC3) is found on VLDL and CMs and inhibits LPL- and LDL receptor- mediated TRL and remnant clearance (135, 136). In humans, apoC3 is elevated in hyperlipidemia and T2D, thereby increasing plasma residence time of TRLs and their remnants. ApoC3 is an independent risk factor for CVD (137, 138). In patients with familial chylomicronemia syndrome due to homozygous or compound heterozygous *LPL* deficiency, the apoC3 antisense oligonucleotide (ASO) volanesorsen decreased CM-TG and apoB48 suggesting non-LPL mediated clearance (139). Interestingly, while apoC3 is known to increase hepatic VLDL secretion (140, 141), whole-body overexpression of human apoC3 in mice decreased dietary TG appearance in lymph, decreased FA absorption and re-esterification, suggesting intestinal apoC3 inhibition might enhance CM appearance (142). However, apoC3 inhibition by ASO decreased fasting and postprandial plasma TG without altered intestinal fat absorption in mice (143). Whether apoC3 inhibition regulates CM secretion in humans remains to be established. In humans, a loss-of-function apoC3 variant associated with decreased TG and CVD protection had no effect on apoB concentration (144), perhaps suggesting no effect on CM secretion.

Several angiopoietin-like proteins (ANGPTLs), namely ANGPTL3, ANGPTL4, and ANGPTL8, have been identified as inhibitors of LPL activity resulting in increased plasma TG (145) (Figure 1, #8). In mice and humans, deletion or loss-of-function in any of these genes reduced plasma TG while overexpression increased plasma TG (145). ANGPTL3 ASO reduced VLDL cholesterol (146), however its effects on intestinal CM secretion have not been established. ANGPTL4 expression in adipose tissue is increased upon fasting to inhibit adipose LPL activity, redirecting TG to other tissues for oxidation (147). Upon high-fat feeding, enterocyte ANGPTL4 expression is increased possibly to inhibit pancreatic lipase and slow FA uptake into enterocytes to match FA uptake with TG secretion (148). Whether intestinal ANGPTL4 inhibition alters CM secretion remains to be investigated. Inactivating ANGPTL4 variants in humans are associated with reduced risk of coronary disease (149), however ANGPTL4 inhibition by monoclonal antibodies in mice and non-human primates was associated with lymphadenopathy (150). Whether this adverse response would be present in humans is unknown, although subjects with inactivating ANGPTL4 variants do not exhibit lymphatic abnormalities (150). In humans ANGPTL8 is exclusively enriched in the liver (151) and thus it is unlikely to modulate CM secretion. In mice its expression is enhanced by insulin signaling through the insulin responsive transcription factor liver X receptor α to promote VLDL secretion (152). Of these ANGPTL peptides, ANGPTL4 is the only one known to be expressed in the intestine and thus may serve as a therapeutic target to modulate CM secretion, however adverse effects may preclude its further development. Whether ANGPTL3 or ANGPTL8 inhibition could modulate CM secretion remains to be determined.

## Conclusions

Diabetic dyslipidemia is characterized by increased fasting and postprandial TGs, packaged within apoB-containing lipoproteins, many of which are considered to be directly implicated in promoting atherosclerotic CVD. Worsening IR is correlated with increases in circulating CMs arising from increased production and decreased clearance. Increased intestinal CM production in insulin resistant states can be attributed to increased supply of lipogenic substrates and resistance to key modulatory signals that results in altered expression and activity of lipogenic and secretory pathways. Therapeutic strategies developed thus far to directly target the gut have been limited by GI and other adverse events, precluding their widespread use. Approved lipid and glucose modifying therapies such as statins, fibrates, ezetimibe, SGLT2 inhibitors, metformin, and GLP-1 receptor agonists have also been shown to affect intestinal CM production. This is a very fertile area for drug development, but major challenges remain in improving the tolerability of agents that target gut lipid handling and CM secretion before these agents can be deployed to curb CVD risk in those with and without T2D.

## Author Contributions

PS and GL wrote the manuscript. CX and AN edited the manuscript.

### Conflict of Interest

GL holds the Drucker Family Chair in Diabetes Research and the Sun Life Financial Chair in Diabetes. The remaining authors declare that the research was conducted in the absence of any commercial or financial relationships that could be construed as a potential conflict of interest.

## Abbreviations

CM: chylomicron
CVD: cardiovascular disease
DGAT: diacylglycerol acyltransferase
DNL: *de novo* lipogenesis
ER: endoplasmic reticulum
FA: fatty acid
FABP: fatty acid binding protein
FAT/CD36: fatty acid translocase/cluster of differentiation 36
GI: gastrointestinal
GLP-1: glucagon-like peptide-1
GLP-2: glucagon-like peptide-2
HoFH: homozygous familial hypercholesterolemia
HOMA-IR: homeostatic model assessment of insulin resistance
IR: insulin resistance
LPL: lipoprotein lipase
MAG: monoacylglycerol
MAPK: mitogen-activated protein kinase
MGAT: monoacylglycerol acyltransferase
MTP: microsomal triglyceride transfer protein
NPC1L1: Niemann-Pick C1-like 1 protein
PCSK9: proprotein convertase subtilisin/kexin type
PPARα: peroxisome proliferator-activated receptor alpha
SGLT: Sodium-glucose co-transporter
SNARE: soluble N-ethylmaleimide-sensitive fusion protein attachment protein receptors
SREBP: sterol regulatory element binding protein
T2D: type 2 diabetes
TG: triglyceride
TRL: triglyceride-rich lipoprotein
VLDL: very-low density lipoprotein.

## References

[1] Lewis GF Xiao C Hegele RA. Hypertriglyceridemia in the genomic era: a new paradigm. Endocr Rev. (2015) 36:131–47. 10.1210/er.2014-1062 25554923

[2] Proctor SD Vine DF Mamo JC Arterial retention of apolipoprotein B48- and B100-containing lipoproteins in atherogenesis. Curr Opin Lipidol. (2002) 13:461–70. 10.1097/00041433-200210000-00001 12352009

[3] Hassing HC Surendran RP Mooij HL Stroes ES Nieuwdorp M Dallinga-Thie GM. Pathophysiology of hypertriglyceridemia. Biochim Biophys Acta. (2012) 1821:826–32. 10.1016/j.bbalip.2011.11.010 22179026

[4] Karpe F. Postprandial lipoprotein metabolism and atherosclerosis. J Intern Med. (1999) 246:341–55. 10.1046/j.1365-2796.1999.00548.x 10583705

[5] Brunzell JD Hazzard WR Porte D Bierman EL. Evidence for a common, saturable, triglyceride removal mechanism for chylomicrons and very low density lipoproteins in man. J Clin Invest. (1973) 52:1578–85. 10.1172/JCI107334 4352459 PMC302428

[6] Cooper AD. Hepatic uptake of chylomicron remnants. J Lipid Res. (1997) 38:2173–92. 9392416

[7] Pang J Chan DC Watts GF. Origin and therapy for hypertriglyceridaemia in type 2 diabetes. World J Diabetes. (2014) 5:165. 10.4239/wjd.v5.i2.165 24748930 PMC3990315

[8] Laatsch A Merkel M Talmud PJ Grewal T Beisiegel U Heeren J. Insulin stimulates hepatic low density lipoprotein receptor-related protein 1 (LRP1) to increase postprandial lipoprotein clearance. Atherosclerosis. (2009) 204:105–11. 10.1016/j.atherosclerosis.2008.07.046 18834984

[9] Dash S Xiao C Morgantini C Lewis GF. New insights into the regulation of chylomicron production. Annu Rev Nutr. (2015) 35:265–94. 10.1146/annurev-nutr-071714-034338 25974693

[10] Hussain MM. A proposed model for the assembly of chylomicrons. Atherosclerosis. (2000) 148:1–15. 10.1016/S0021-9150(99)00397-4 10580165

[11] Khatun I Clark RW Vera NB Kou K Erion DM Coskran T. Characterization of a novel intestinal glycerol-3-phosphate acyltransferase pathway and its role in lipid homeostasis. J Biol Chem. (2016) 291:2602–15. 10.1074/jbc.M115.683359 26644473 PMC4742731

[12] Kohan AB Wang F Li X Bradshaw S Yang Q Caldwell JL. Apolipoprotein A-IV regulates chylomicron metabolism–mechanism and function. Am J Physiol Gastrointest Liver Physiol. (2012) 302:G628–36. 10.1152/ajpgi.00225.2011 22207575 PMC3311309

[13] Tso P Balint JA. Formation and transport of chylomicrons by enterocytes to the lymphatics. Am J Physiol Gastrointest Liver Physiol. (1986) 250:G715–26. 10.1152/ajpgi.1986.250.6.G715 3521320

[14] Zhou A Qu J Liu M Tso P. The role of interstitial matrix and the lymphatic system in gastrointestinal lipid and lipoprotein metabolism. Front Physiol. (2020) 11:4. 10.3389/fphys.2020.00004 32038309 PMC6987427

[15] Xiao C Stahel P Lewis GF. Regulation of chylomicron secretion: focus on post-assembly mechanisms. Cell Mol Gastroenterol Hepatol. (2019) 7:487–501. 10.1016/j.jcmgh.2018.10.015 30819663 PMC6396431

[16] Xiao C Hsieh J Adeli K Lewis GF. Gut-liver interaction in triglyceride-rich lipoprotein metabolism. Am J Physiol Endocrinol Metab. (2011) 301:E429–46. 10.1152/ajpendo.00178.2011 21693689

[17] Robertson MD Parkes M Warren BF Ferguson DJP Jackson KG Jewell DP. Mobilisation of enterocyte fat stores by oral glucose in humans. Gut. (2003) 52:834–9. 10.1136/gut.52.6.834 12740339 PMC1773679

[18] Xiao C Stahel P Carreiro AL Hung YH Dash S Bookman I. Oral glucose mobilizes triglyceride stores from the human intestine. Cell Mol Gastroenterol Hepatol. (2019) 7:313–37. 10.1016/j.jcmgh.2018.10.002 30704982 PMC6357697

[19] Chavez–Jauregui RN Mattes RD Parks EJ Dynamics of fat absorption and effect of sham feeding on postprandial lipemia. Gastroenterology. (2010) 139:1538–48. 10.1053/j.gastro.2010.05.002 20493191 PMC2948783

[20] Mulvihill EE. Regulation of intestinal lipid and lipoprotein metabolism by the proglucagon-derived peptides glucagon like peptide 1 and glucagon like peptide 2. Curr Opin Lipidol. (2018) 29:95–103. 10.1097/MOL.0000000000000495 29432213 PMC5882252

[21] Leon-Acuña A Alcala-Diaz JF Delgado-Lista J Torres-Peña JD Lopez-Moreno J Camargo A. Hepatic insulin resistance both in prediabetic and diabetic patients determines postprandial lipoprotein metabolism: from the CORDIOPREV study. Cardiovasc Diabetol. (2016) 15:68. 10.1186/s12933-016-0380-y 27095446 PMC4837552

[22] Duez H Lamarche B Uffelman KD Valero R Cohn JS Lewis GF. Hyperinsulinemia is associated with increased production rate of intestinal apolipoprotein b-48–containing lipoproteins in humans. Arterioscler Thromb Vasc Biol. (2006) 26:1357–63. 10.1161/01.ATV.0000222015.76038.14 16614317

[23] Couture P Tremblay AJ Kelly I Lemelin V Droit A Lamarche B. Key intestinal genes involved in lipoprotein metabolism are downregulated in dyslipidemic men with insulin resistance. J Lipid Res. (2014) 55:128–37. 10.1194/jlr.M040071 24142110 PMC3927480

[24] Shojaee-Moradie F Ma Y Lou S Hovorka R Umpleby AM. Prandial hypertriglyceridemia in metabolic syndrome is due to an overproduction of both chylomicron and VLDL triacylglycerol. Diabetes. (2013) 62:4063–69. 10.2337/db13-0935 23990358 PMC3837057

[25] Hogue J-C Lamarche B Tremblay AJ Bergeron J Gagné C Couture P. Evidence of increased secretion of apolipoprotein B-48-containing lipoproteins in subjects with type 2 diabetes. J Lipid Res. (2007) 48:1336–42. 10.1194/jlr.M600548-JLR200 17337758

[26] Duvillard L Pont F Florentin E Galland-Jos C Gambert P Vergès B. Metabolic abnormalities of apolipoprotein B-containing lipoproteins in non-insulin-dependent diabetes: a stable isotope kinetic study. Eur J Clin Invest. (2000) 30:685–94. 10.1046/j.1365-2362.2000.00755.x 10964160

[27] Larsen MA Goll R Lekahl S Moen OS Florholmen J. Delayed clearance of triglyceride-rich lipoproteins in young, healthy obese subjects. Clin Obes. (2015) 5:349–57. 10.1111/cob.12118 26469529 PMC5111784

[28] Pavlic M Xiao C Szeto L Patterson BW Lewis GF. Insulin acutely inhibits intestinal lipoprotein secretion in humans in part by suppressing plasma free fatty acids. Diabetes. (2010) 59:580–7. 10.2337/db09-1297 20028946 PMC2828667

[29] Leung N Naples M Uffelman K Szeto L Adeli K Lewis GF. Rosiglitazone improves intestinal lipoprotein overproduction in the fat-fed Syrian Golden hamster, an animal model of nutritionally-induced insulin resistance. Atherosclerosis. (2004) 174:235–41. 10.1016/j.atherosclerosis.2004.02.005 15136053

[30] Federico LM Naples M Taylor D Adeli K. Intestinal insulin resistance and aberrant production of apolipoprotein B48 lipoproteins in an animal model of insulin resistance and metabolic dyslipidemia evidence for activation of protein tyrosine phosphatase-1b, extracellular signal-related kinase, and sterol regulatory element-binding protein-1c in the fructose-fed hamster intestine. Diabetes. (2006) 55:1316–26. 10.2337/db04-1084 16644688

[31] Haidari M Leung N Mahbub F Uffelman KD Kohen-Avramoglu R Lewis GF. Fasting and postprandial overproduction of intestinally derived lipoproteins in an animal model of insulin resistance. J Biol Chem. (2002) 277:31646–55. 10.1074/jbc.M200544200 12070142

[32] Tran TTT Postal BG Demignot S Ribeiro A Osinski C Pais de Barros J-P. Short term palmitate supply impairs intestinal insulin signaling via ceramide production. J Biol Chem. (2016) 291:16328–38. 10.1074/jbc.M115.709626 27255710 PMC4965580

[33] Larsen PJ Tennagels N. On ceramides, other sphingolipids and impaired glucose homeostasis. Mol Metab. (2014) 3:252–60. 10.1016/j.molmet.2014.01.011 24749054 PMC3986510

[34] Hsieh J Hayashi AA Webb J Adeli K. Postprandial dyslipidemia in insulin resistance: mechanisms and role of intestinal insulin sensitivity. Atheroscler Suppl. (2008) 9:7–13. 10.1016/j.atherosclerosissup.2008.05.011 18653387

[35] Zoltowska M Ziv E Delvin E Sinnett D Kalman R Garofalo C. Cellular aspects of intestinal lipoprotein assembly in *Psammomys obesus*: a model of insulin resistance and type 2 diabetes. Diabetes. (2003) 52:2539–45. 10.2337/diabetes.52.10.2539 14514638

[36] Veilleux A Grenier É Marceau P Carpentier AC Richard D Levy E. Intestinal lipid handling: evidence and implication of insulin signaling abnormalities in human obese subjects. Arterioscler Thromb Vasc Biol. (2014) 34:644–53. 10.1161/ATVBAHA.113.302993 24407032

[37] Phillips C Mullan K Owens D Tomkin GH. Intestinal microsomal triglyceride transfer protein in type 2 diabetic and non-diabetic subjects: the relationship to triglyceride-rich postprandial lipoprotein composition. Atherosclerosis. (2006) 187:57–64. 10.1016/j.atherosclerosis.2005.08.020 16183064

[38] Duez H Lamarche B Valéro R Pavlic M Proctor S Xiao C Szeto L Patterson BW Lewis GF. Both intestinal and hepatic lipoprotein production are stimulated by an acute elevation of plasma free fatty acids in humans. Circulation. (2008) 117:2369–76. 10.1161/CIRCULATIONAHA.107.739888 18443237 PMC3874779

[39] Orshov C Holst JJ Knuhtsen S Baldissera FGA Poulsen SS Nielsen OV Glucagon-like peptides glp-1 and glp-2, predicted products of the glucagon gene, are secreted separately from pig small intestine but not pancreas. Endocrinology. (1986) 119:1467–75. 10.1210/endo-119-4-1467 3530719

[40] Baggio LL Drucker DJ. Clinical endocrinology and metabolism. Glucagon-like peptide-1 and glucagon-like peptide-2. Best Pract Res Clin Endocrinol Metab. (2004) 18:531–54. 10.1016/j.beem.2004.08.001 15533774

[41] Hein GJ Baker C Hsieh J Farr S Adeli K. GLP-1 and GLP-2 as yin and yang of intestinal lipoprotein production: Evidence for predominance of GLP-2-stimulated postprandial lipemia in normal and insulin-resistant states. Diabetes. (2013) 62:373–81. 10.2337/db12-0202 23028139 PMC3554391

[42] Xiao C Bandsma RHJ Dash S Szeto L Lewis GF. Exenatide, a glucagon-like peptide-1 receptor agonist, acutely inhibits intestinal lipoprotein production in healthy humans. Arterioscler Thromb Vasc Biol. (2012) 32:1513–9. 10.1161/ATVBAHA.112.246207 22492091

[43] Hsieh J Longuet C Maida A Bahrami J Xu E Baker CL. Glucagon-like peptide-2 increases intestinal lipid absorption and chylomicron production via CD36. Gastroenterology. (2009) 137:997–1005. 10.1053/j.gastro.2009.05.051 19482026

[44] Hsieh J Trajcevski KE Farr SL Baker CL Lake EJ Taher J. Glucagon-like peptide 2 (GLP-2) stimulates postprandial chylomicron production and postabsorptive release of intestinal triglyceride storage pools via induction of nitric oxide signaling in male hamsters and mice. Endocrinology. (2015) 156:3538–47. 10.1210/EN.2015-1110 26132919

[45] Dash S Xiao C Morgantini C Connelly PW Patterson BW Lewis GF. Glucagon-like peptide-2 regulates release of chylomicrons from the intestine. Gastroenterology. (2014) 147:1275–84. 10.1053/j.gastro.2014.08.037 25173752 PMC4316201

[46] Stahel P Xiao C Davis X Tso P Lewis GF. Glucose and GLP-2 (glucagon-like peptide-2) mobilize intestinal triglyceride by distinct mechanisms. Arterioscler Thromb Vasc Biol. (2019) 39:1565–73. 10.1161/ATVBAHA.119.313011 31294621 PMC6657524

[47] Anini Y Brubaker PL. Role of leptin in the regulation of glucagon-like peptide-1 secretion. Diabetes. (2003) 52:252–9. 10.2337/diabetes.52.2.252 12540594

[48] Cammisotto PG Levy É Bukowiecki LJ Bendayan M. Cross-talk between adipose and gastric leptins for the control of food intake and energy metabolism. Prog Histochem Cytochem. (2010) 45:143–200. 10.1016/j.proghi.2010.06.001 20621336

[49] Iqbal J Li X Chang BH Chan L Schwartz GJ Chua SC. An intrinsic gut leptin-melanocortin pathway modulates intestinal microsomal triglyceride transfer protein and lipid absorption. J Lipid Res. (2010) 51:1929–42. 10.1194/jlr.M005744 20164094 PMC2882750

[50] Nogueira J-P Maraninchi M Béliard S Padilla N Duvillard L Mancini J. Absence of acute inhibitory effect of insulin on chylomicron production in type 2 diabetes. Arterioscler Thromb Vasc Biol. (2012) 32:1039–44. 10.1161/ATVBAHA.111.242073 22308041

[51] Mäkinen J Hannukainen JC Karmi A Immonen HM Soinio M Nelimarkka L. Obesity-associated intestinal insulin resistance is ameliorated after bariatric surgery. Diabetologia. (2015) 58:1055–62. 10.1007/s00125-015-3501-3 25631620 PMC4392118

[52] Griffo E Nosso G Lupoli R Cotugno M Saldalamacchia G Vitolo G. Early improvement of postprandial lipemia after bariatric surgery in obese type 2 diabetic patients. Obes Surg. (2014) 24:765–70. 10.1007/s11695-013-1148-z 24374941

[53] Padilla N Maraninchi M Béliard S Berthet B Nogueira J-P Wolff E. Effects of bariatric surgery on hepatic and intestinal lipoprotein particle metabolism in obese, nondiabetic humans. Arterioscler Thromb Vasc Biol. (2014) 34:2330–7. 10.1161/ATVBAHA.114.303849 25104797

[54] Malmström R Packard CJ Caslake M Bedford D Stewart P Yki-Järvinen H. Defective regulation of triglyceride metabolism by insulin in the liver in NIDDM. Diabetologia. (1997) 40:454–62. 10.1007/s001250050700 9112023

[55] Xiao C Dash S Morgantini C Lewis GF. Novel role of enteral monosaccharides in intestinal lipoprotein production in healthy humans. Arterioscler Thromb Vasc Biol. (2013) 33:1056–62. 10.1161/ATVBAHA.112.300769 23471231

[56] Xiao C Dash S Morgantini C Lewis GF. Intravenous glucose acutely stimulates intestinal lipoprotein secretion in healthy humans. Atheroscler Thromb Vasc Biol. (2016) 36:1457–63. 10.1161/ATVBAHA.115.307044 27150393

[57] Phillips C Madigan C Owens D Collins P Tomkin GH. Defective chylomicron synthesis as a cause of delayed particle clearance in diabetes? Int J Exp Diabetes Res. (2002) 3:171–8. 10.1080/15604280214277 12458658 PMC2478583

[58] Gutierrez-Repiso C Rodriguez-Pacheco F Garcia-Arnes J Valdes S Gonzalo M Soriguer F. The expression of genes involved in jejunal lipogenesis and lipoprotein synthesis is altered in morbidly obese subjects with insulin resistance. Lab Invest. (2015) 95:1409–17. 10.1038/labinvest.2015.115 26367490

[59] Verges BL Vaillant G Goux A Lagrost L Brun JM Gambert P. Apolipoprotein A-IV levels and phenotype distribution in NIDDM. Diabetes Care. (1994) 17:810–7. 10.2337/diacare.17.8.810 7956623

[60] Dai Y Shen Y Li QR Ding FH Wang XQ Liu HJ. Glycated apolipoprotein A-IV induces atherogenesis in patients with CAD in type 2 diabetes. J Am Coll Cardiol. (2017) 70:2006–19. 10.1016/j.jacc.2017.08.053 29025558

[61] Martins I Mortimer B Miller J Redgrave T. Effects of particle size and number on the plasma clearance of chylomicrons and remnants. J Lipid Res. (1996) 37:2696–705. 9017520

[62] Qi K Al-Haideri M Seo T Carpentier YA Deckelbaum RJ. Effects of particle size on blood clearance and tissue uptake of lipid emulsions with different triglyceride compositions. J Parenter Enter Nutr. (2003) 27:58–64. 10.1177/014860710302700158 12549600

[63] Qu J Ko C-W Tso P Bhargava A. Apolipoprotein A-IV: a multifunctional protein involved in protection against atherosclerosis and diabetes. Cells. (2019) 8:319. 10.3390/cells8040319 30959835 PMC6523623

[64] Colhoun HM Betteridge DJ Durrington PN Hitman GA Neil HAW Livingstone SJ. Primary prevention of cardiovascular disease with atorvastatin in type 2 diabetes in the Collaborative Atorvastatin Diabetes Study (CARDS): multicentre randomised placebo-controlled trial. Lancet. (2004) 364:685–96. 10.1016/S0140-6736(04)16895-5 15325833

[65] Pyorälä K Pedersen TR Kjekshus J Faergeman O Olsson AG Thorgeirsson G. Cholesterol lowering with simvastatin improves prognosis of diabetic patients with coronary heart disease: a subgroup analysis of the Scandinavian Simvastatin Survival Study (4S). Diabetes Care. (1997) 20:614–20. 10.2337/diacare.20.4.614 9096989

[66] Battula SB Fitzsimons O Moreno S Owens D Collins P Johnson A. Postprandial apolipoprotein B48– and B100–containing lipoproteins in type 2 diabetes: do statins have a specific effect on triglyceride metabolism? Metab Clin Exp. (2000) 49:1049–54. 10.1053/meta.2000.7744 10954025

[67] Chan DC Watts GF Barrett PHR Martins IJ James AP Mamo JCL. Effect of atorvastatin on chylomicron remnant metabolism in visceral obesity: a study employing a new stable isotope breath test. J Lipid Res. (2002) 43:706–12. 11971941

[68] Chan DC Watts GF Somaratne R Wasserman SM Scott R Barrett PHR. Comparative effects of PCSK9 (Proprotein Convertase Subtilisin/Kexin Type 9) inhibition and statins on postprandial triglyceride-rich lipoprotein metabolism. Arterioscler Thromb Vasc Biol. (2018) 38:1644–55. 10.1161/ATVBAHA.118.310882 29880491 PMC6039422

[69] Tremblay AJ Lamarche B Lemelin V Hoos L Benjannet S Seidah NG. Atorvastatin increases intestinal expression of NPC1L1 in hyperlipidemic men. J Lipid Res. (2011) 52:558–565. 10.1194/jlr.M011080 21123766 PMC3035692

[70] Bozzetto L Annuzzi G Corte GD Patti L Cipriano P Mangione A. Ezetimibe beneficially influences fasting and postprandial triglyceride-rich lipoproteins in type 2 diabetes. Atherosclerosis. (2011) 217:142–8. 10.1016/j.atherosclerosis.2011.03.012 21481394

[71] Ruggenenti P Cattaneo D Rota S Iliev I Parvanova A Diadei O. Effects of combined ezetimibe and simvastatin therapy as compared with simvastatin alone in patients with type 2 diabetes: a prospective randomized double-blind clinical trial. Diabetes Care. (2010) 33:1954–6. 10.2337/dc10-0320 20566677 PMC2928341

[72] Jun M Foote C Lv J Neal B Patel A Nicholls SJ. Effects of fibrates on cardiovascular outcomes: a systematic review and meta-analysis. Lancet. (2010) 375:1875–84. 10.1016/S0140-6736(10)60656-3 20462635

[73] Elam M Lovato L Ginsberg H. The ACCORD-Lipid study: implications for treatment of dyslipidemia in Type 2 diabetes mellitus. Clin Lipidol. (2011) 6:9–20. 10.2217/clp.10.84 26207146 PMC4509601

[74] Contreras AV Torres N Tovar AR. PPAR-α as a key nutritional and environmental sensor for metabolic adaptation. Adv Nutr. (2013) 4:439–52. 10.3945/an.113.003798 23858092 PMC3941823

[75] Arai H Yamashita S Yokote K Araki E Suganami H Ishibashi S. Efficacy and safety of pemafibrate versus fenofibrate in patients with high triglyceride and low HDL cholesterol levels: a multicenter, placebo-controlled, double-blind, randomized trial. J Atheroscler Thromb. (2018) 25:521–38. 10.5551/jat.44412 29628483 PMC6005227

[76] Ida S Kaneko R Murata K. Efficacy and safety of pemafibrate administration in patients with dyslipidemia: a systematic review and meta-analysis. Cardiovasc Diabetol. (2019) 18. 10.1186/s12933-019-0845-x 30898163 PMC6429757

[77] Araki E Yamashita S Arai H Yokote K Satoh J Inoguchi T. Efficacy and safety of pemafibrate in people with type 2 diabetes and elevated triglyceride levels: 52-week data from the PROVIDE study. Diabetes Obes Metab. (2019) 21:1737–44. 10.1111/dom.13686 30830727 PMC6617746

[78] Hennuyer N Duplan I Paquet C Vanhoutte J Woitrain E Touche V. The novel selective PPARα modulator (SPPARMα) pemafibrate improves dyslipidemia, enhances reverse cholesterol transport and decreases inflammation and atherosclerosis. Atherosclerosis. (2016) 249:200–8. 10.1016/j.atherosclerosis.2016.03.003 27108950

[79] Takei K Nakagawa Y Wang Y Han S Satoh A Sekiya M. Effects of K-877, a novel selective PPARα modulator, on small intestine contribute to the amelioration of hyperlipidemia in low-density lipoprotein receptor knockout mice. J Pharmacol Sci. (2017) 133:214–22. 10.1016/j.jphs.2017.02.003 28366492

[80] Pradhan AD Paynter NP Everett BM Glynn RJ Amarenco P Elam M. Rationale and design of the pemafibrate to reduce cardiovascular outcomes by reducing triglycerides in patients with diabetes (PROMINENT) study. Am Heart J. (2018) 206:80–93. 10.1016/j.ahj.2018.09.011 30342298

[81] Lagace TA. PCSK9 and LDLR degradation: regulatory mechanisms in circulation and in cells. Curr Opin Lipidol. (2014) 25:387–93. 10.1097/MOL.0000000000000114 25110901 PMC4166010

[82] Le May C Kourimate S Langhi C Chétiveaux M Jarry A Comera C. Proprotein Convertase Subtilisin Kexin Type 9 null mice are protected from postprandial triglyceridemia. Arterioscler Thromb Vasc Biol. (2009) 29:684–90. 10.1161/ATVBAHA.108.181586 19265033

[83] Rashid S Tavori H Brown PE Linton MF He J Giunzioni I. Proprotein Convertase subtilisin kexin Type 9 promotes intestinal overproduction of triglyceride-rich apolipoprotein B lipoproteins through both low-density lipoprotein receptor–dependent and –independent mechanisms. Circulation. (2014) 130:431–41. 10.1161/CIRCULATIONAHA.113.006720 25070550 PMC4115295

[84] Reyes-Soffer G Pavlyha M Ngai C Thomas T Holleran S Ramakrishnan R. Effects of PCSK9 inhibition with alirocumab on lipoprotein metabolism in healthy humans. Circulation. (2017) 135:352–62. 10.1161/CIRCULATIONAHA.116.025253 27986651 PMC5262523

[85] Bailey CJ Wilcock C Scarpello JHB. Metformin and the intestine. Diabetologia. (2008) 51:1552–3. 10.1007/s00125-008-1053-5 18528677

[86] Bonora E Cigolini M Bosello O Zancanaro C Capretti L Zavaroni I. Lack of effect of intravenous metformin on plasma concentrations of glucose, insulin, C-peptide, glucagon and growth hormone in non-diabetic subjects. Curr Med Res Opin. (1984) 9:47–51. 10.1185/03007998409109558 6373159

[87] Jeppesen J Zhou MY Chen YD Reaven GM. Effect of metformin on postprandial lipemia in patients with fairly to poorly controlled NIDDM. Diabetes Care. (1994) 17:1093–99. 10.2337/diacare.17.10.1093 7821127

[88] Napolitano A Miller S Nicholls AW Baker D Van Horn S Thomas E. Novel gut-based pharmacology of metformin in patients with type 2 diabetes mellitus. PLoS ONE. (2014) 9:e100778. 10.1371/journal.pone.0100778 24988476 PMC4079657

[89] Preiss D Dawed A Welsh P Heggie A Jones AG Dekker J. Sustained influence of metformin therapy on circulating glucagon-like peptide-1 levels in individuals with and without type 2 diabetes. Diabetes Obes Metab. (2017) 19:356–363. 10.1111/dom.12826 27862873 PMC5330429

[90] Sato D Morino K Ogaku S Tsuji A Nishimura K Sekine O. Efficacy of metformin on postprandial plasma triglyceride concentration by administration timing in patients with type 2 diabetes mellitus: a randomized cross-over pilot study. J Diabetes Investig. (2019) 10:1284–90. 10.1111/jdi.13016 30688410 PMC6717824

[91] Hermansen K Bækdal TA Düring M Pietraszek A Mortensen LS Jørgensen H. Liraglutide suppresses postprandial triglyceride and apolipoprotein B48 elevations after a fat-rich meal in patients with type 2 diabetes: a randomized, double-blind, placebo-controlled, cross-over trial. Diabetes Obes Metab. (2013) 15:1040–8. 10.1111/dom.12133 23683069

[92] Hsieh J Longuet C Baker CL Qin B Federico LM Drucker DJ. The glucagon-like peptide 1 receptor is essential for postprandial lipoprotein synthesis and secretion in hamsters and mice. Diabetologia. (2010) 53:552–61. 10.1007/s00125-009-1611-5 19957161

[93] Xiao C Dash S Morgantini C Hegele RA Lewis GF. Pharmacological targeting of the atherogenic dyslipidemia complex: the next frontier in CVD prevention beyond lowering LDL cholesterol. Diabetes. (2016) 65:1767–78. 10.2337/db16-0046 27329952

[94] Meier JJ Rosenstock J Hincelin-Mery A Roy-Duval C Delfolie A Coester H-V Contrasting effects of lixisenatide and liraglutide on postprandial glycemic control, gastric emptying, and safety parameters in patients with type 2 diabetes on optimized insulin glargine with or without metformin: a randomized, open-label trial. Diabetes Care. (2015) 38:1263–73. 10.2337/dc14-1984 25887358

[95] Whyte MB Shojaee-Moradie F Sharaf SE Jackson NC Fielding B Hovorka R. Lixisenatide reduces chylomicron triacylglycerol by increased clearance. J Clin Endocrinol Metab. (2019) 104:359–68. 10.1210/jc.2018-01176 30215735 PMC6300412

[96] Vergès B Duvillard L Pais de Barros JP Bouillet B Baillot-Rudoni S Rouland A. Liraglutide reduces postprandial hyperlipidemia by increasing apoB48 (apolipoprotein B48) catabolism and by reducing apoB48 production in patients with type 2 diabetes mellitus. Arterioscler Thromb Vasc Biol. (2018) 38:2198–206. 10.1161/ATVBAHA.118.310990 30026275

[97] Marso SP Daniels GH Brown-Frandsen K Kristensen P Mann JFE Nauck MA. Liraglutide and cardiovascular outcomes in type 2 diabetes. N Engl J Med. (2016) 375:311–22. 10.1056/NEJMoa1603827 27295427 PMC4985288

[98] Marso SP Bain SC Consoli A Eliaschewitz FG Jódar E Leiter LA. Semaglutide and cardiovascular outcomes in patients with type 2 diabetes. N Engl J Med. (2016) 375:1834–44. 10.1056/NEJMoa1607141 27633186

[99] Wiviott SD Raz I Bonaca MP Mosenzon O Kato ET Cahn A Dapagliflozin and cardiovascular outcomes in type 2 diabetes. N Engl J Med. (2019) 380:347–57. 10.1056/NEJMoa1812389 30415602

[100] Neal B Perkovic V Mahaffey KW Zeeuw D de Fulcher G Erondu N Canagliflozin and cardiovascular and renal events in type 2 diabetes. N Engl J Med. (2017) 377:644–57. 10.1056/NEJMoa1611925 28605608

[101] Zinman B Wanner C Lachin JM Fitchett D Bluhmki E Hantel S. Empagliflozin, cardiovascular outcomes, and mortality in type 2 diabetes. N Engl J Med. (2015) 373:2117–28. 10.1056/NEJMoa1504720 26378978

[102] Kamijo Y Ishii H Yamamoto T Kobayashi K Asano H Miake S. Potential impact on lipoprotein subfractions in type 2 diabetes. Clin Med Insights Endocrinol Diabetes. (2019) 12:117955141986681. 10.1177/1179551419866811 31452606 PMC6696845

[103] Ohgaki R Wei L Yamada K Hara T Kuriyama C Okuda S. Interaction of the Sodium/Glucose Cotransporter. (SGLT) 2 inhibitor Canagliflozin with SGLT1 and SGLT2. J Pharmacol Exp Ther. (2016) 358:94–102. 10.1124/jpet.116.232025 27189972

[104] Polidori D Sha S Mudaliar S Ciaraldi TP Ghosh A Vaccaro N. Canagliflozin lowers postprandial glucose and insulin by delaying intestinal glucose absorption in addition to increasing urinary glucose excretion: results of a randomized, placebo-controlled study. Diabetes Care. (2013) 36:2154–61. 10.2337/dc12-2391 23412078 PMC3714520

[105] Zheng M Yang J Shan C Zhou H Xu Y Wang Y. Effects of 24-week treatment with acarbose on glucagon-like peptide 1 in newly diagnosed type 2 diabetic patients: a preliminary report. Cardiovasc Diabetol. (2013) 12:73. 10.1186/1475-2840-12-73 23642288 PMC3653752

[106] Dobbins RL Greenway FL Chen L Liu Y Breed SL Andrews SM. Selective sodium-dependent glucose transporter 1 inhibitors block glucose absorption and impair glucose-dependent insulinotropic peptide release. Am J Physiol Gastrointest Liver Physiol. (2015) 308:G946–54. 10.1152/ajpgi.00286.2014 25767259

[107] Zambrowicz B Ogbaa I Frazier K Banks P Turnage A Freiman J. Effects of LX4211, a dual sodium-dependent glucose cotransporters 1 and 2 inhibitor, on postprandial glucose, insulin, glucagon-like peptide 1, and peptide tyrosine tyrosine in a dose-timing study in healthy subjects. Clin Ther. (2013) 35:1162–73. 10.1016/j.clinthera.2013.06.011 23911260

[108] Zambrowicz B Ding Z-M Ogbaa I Frazier K Banks P Turnage A. Effects of LX4211, a dual SGLT1/SGLT2 inhibitor, plus sitagliptin on postprandial active GLP-1 and glycemic control in type 2 diabetes. Clin Ther. (2013) 35:273–85. 10.1016/j.clinthera.2013.01.010 23433601

[109] Drew BS Dixon AF Dixon JB. Obesity management: update on orlistat. Vasc Health Risk Manag. (2007) 3:817–21. 18200802 PMC2350121

[110] Hollander PA Elbein SC Hirsch IB Kelley D McGill J Taylor T. Role of orlistat in the treatment of obese patients with type 2 diabetes: a 1-year randomized double-blind study. Diabetes Care. (1998) 21:1288–94. 10.2337/diacare.21.8.1288 9702435

[111] Miles JM Leiter L Hollander P Wadden T Anderson JW Doyle M. Effect of orlistat in overweight and obese patients with type 2 diabetes treated with metformin. Diabetes Care. (2002) 25:1123–8. 10.2337/diacare.25.7.1123 12087008

[112] Shi Y Cheng D. Beyond triglyceride synthesis: the dynamic functional roles of MGAT and DGAT enzymes in energy metabolism. Am J Physiol Endocrinol Metab. (2009) 297:E10–8. 10.1152/ajpendo.90949.2008 19116371 PMC3735925

[113] Take K Mochida T Maki T Satomi Y Hirayama M Nakakariya M. Pharmacological inhibition of monoacylglycerol o-acyltransferase 2 improves hyperlipidemia, obesity, and diabetes by change in intestinal fat utilization. PLoS ONE. (2016) 11:e0150976. 10.1371/journal.pone.0150976 26938273 PMC4777574

[114] Adachi R Ishii T Matsumoto S Satou T Sakamoto J Kawamoto T. Discovery of human intestinal MGAT inhibitors using high-throughput mass spectrometry. SLAS Discov Adv Life Sci RD. (2017) 22:360–5. 10.1177/1087057116673181 28328319

[115] Ables GP Yang KJZ Vogel S Hernandez-Ono A Yu S Yuen JJ. Intestinal DGAT1 deficiency reduces postprandial triglyceride and retinyl ester excursions by inhibiting chylomicron secretion and delaying gastric emptying. J Lipid Res. (2012) 53:2364–79. 10.1194/jlr.M029041 22911105 PMC3466005

[116] Liu J McLaren DG Chen D Kan Y Stout SJ Shen X. Potential mechanism of enhanced postprandial glucagon-like peptide-1 release following treatment with a diacylglycerol acyltransferase 1 inhibitor. Pharmacol Res Perspect. (2015) 3:e00193. 10.1002/prp2.193 27022467 PMC4777249

[117] Liu J Gorski JN Gold SJ Chen D Chen S Forrest G. Pharmacological inhibition of diacylglycerol acyltransferase 1 reduces body weight and modulates gut peptide release–potential insight into mechanism of action. Obes Silver Spring Md. (2013) 21:1406–15. 10.1002/oby.20193 23671037

[118] Meyers CD Tremblay K Amer A Chen J Jiang L Gaudet D. Effect of the DGAT1 inhibitor pradigastat on triglyceride and apoB48 levels in patients with familial chylomicronemia syndrome. Lipids Health Dis. (2015) 14. 10.1186/s12944-015-0006-5 25889044 PMC4337059

[119] Denison H Nilsson C Löfgren L Himmelmann A Mårtensson G Knutsson M. Diacylglycerol acyltransferase 1 inhibition with AZD7687 alters lipid handling and hormone secretion in the gut with intolerable side effects: a randomized clinical trial. Diabetes Obes Metab. (2014) 16:334–43. 10.1111/dom.12221 24118885

[120] Haas JT Winter HS Lim E Kirby A Blumenstiel B DeFelice M. DGAT1 mutation is linked to a congenital diarrheal disorder. J Clin Invest. (2012) 122:4680–4. 10.1172/JCI64873 23114594 PMC3533555

[121] Kjems L Meyers C Thuren T Diacylglycerol acyltransferase 1 (DGAT1) inhibition as a metabolic regulator: clinical benefits of pradigastat in obese patients with type 2 diabetes. J Clin Lipidol. (2014) 8:301–2. 10.1016/j.jacl.2014.02.021

[122] Maciejewski BS Manion TB Steppan CM. Pharmacological inhibition of diacylglycerol acyltransferase-1 and insights into postprandial gut peptide secretion. World J Gastrointest Pathophysiol. (2017) 8:161–75. 10.4291/wjgp.v8.i4.161 29184702 PMC5696614

[123] Velliquette RA Grann K Missler SR Patterson J Hu C Gellenbeck KW. Identification of a botanical inhibitor of intestinal diacylglyceride acyltransferase 1 activity via in vitro screening and a parallel, randomized, blinded, placebo-controlled clinical trial. Nutr Metab. (2015) 12:27. 10.1186/s12986-015-0025-2 26246845 PMC4526202

[124] Kumar S Tirunagaru V Ariazi J Awasthi A Jayaraman V Arumugam P A novel acyl-CoA: diacylglycerol acyltransferase 1. (DGAT1) inhibitor, GSK2973980A, inhibits postprandial triglycerides and reduces body weight in a rodent diet-induced obesity model. J Pharm Res Int. (2017) 18:1–15. 10.9734/JPRI/2017/36835

[125] Villanueva CJ Monetti M Shih M Zhou P Watkins SM Bhanot S. Specific role for acyl CoA: diacylglycerol acyltransferase 1. (Dgat1) in hepatic steatosis due to exogenous fatty acids. Hepatology. (2009) 50:434–42. 10.1002/hep.22980 19472314 PMC3097135

[126] Choi CS Savage DB Kulkarni A Yu XX Liu Z-X Morino K Suppression of diacylglycerol acyltransferase-2 (*DGAT2*), but not *DGAT1*, with antisense oligonucleotides reverses diet-induced hepatic steatosis and insulin resistance. J Biol Chem. (2007) 282:22678–88. 10.1074/jbc.M704213200 17526931

[127] Sacks FM Stanesa M Hegele RA. Severe hypertriglyceridemia with pancreatitis: Thirteen years' treatment with lomitapide. JAMA Intern Med. (2014) 174:443–7. 10.1001/jamainternmed.2013.13309 24366202

[128] Panno MD Cefalù AB Averna MR Lomitapide: a novel drug for homozygous familial hypercholesterolemia. Clin Lipidol. (2014) 9:19–32. 10.2217/clp.13.74

[129] Lin M Zhao S Shen L Xu D. Potential approaches to ameliorate hepatic fat accumulation seen with MTP inhibition. Drug Saf. (2014) 37:213–24. 10.1007/s40264-014-0147-x 24627311

[130] Mera Y Odani N Kawai T Hata T Suzuki M Hagiwara A Pharmacological characterization of diethyl-2-({3-dimethylcarbamoyl-4-[(4′-trifluoromethylbiphenyl-2-carbonyl)amino]phenyl}acetyloxymethyl)-2-phenylmalonate. (JTT-130), an intestine-specific inhibitor of microsomal triglyceride transfer protein. J Pharmacol Exp Ther. (2011) 336:321–7. 10.1124/jpet.110.173807 20974698

[131] Mera Y Kawai T Ogawa N Odani N Sasase T Miyajima K. JTT-130, a novel intestine-specific inhibitor of microsomal triglyceride transfer protein, ameliorates lipid metabolism and attenuates atherosclerosis in hyperlipidemic animal models. J Pharmacol Sci. (2015) 129:169–76. 10.1016/j.jphs.2015.10.004 26598005

[132] Kim E Campbell S Schueller O Wong E Cole B Kuo J. A small-molecule inhibitor of enterocytic microsomal triglyceride transfer protein, SLx-4090, biochemical, pharmacodynamic, pharmacokinetic and safety profile. J Pharmacol Exp Ther. (2011) 337:775–85. 10.1124/jpet.110.177527 21406547

[133] Sweetnam P Kim E Yang Y Campbell S SLx-4090: A novel, intestinal-specific mtp inhibitor for lowering plasma triglyceride and cholesterol levels in dyslipidemia. Am J Gastroenterol. (2005) 100:S100–1. 10.14309/00000434-200509001-00238

[134] Tong W Paradise E Kim E Didio K Schueller O Ferkany J Clinical investigations of SLx-4090 in combination with metformin in type 2 diabetics. In: 70th Scientific Sessions, Orlando, FL (2010).

[135] Windler E Havel RJ. Inhibitory effects of C apolipoproteins from rats and humans on the uptake of triglyceride-rich lipoproteins and their remnants by the perfused rat liver. J Lipid Res. (1985) 26:556–65. 4020294

[136] Mahley RW Innerarity TL Rall SC Weisgraber KH. Plasma lipoproteins: apolipoprotein structure and function. J Lipid Res. (1984) 25:1277–94. 6099394

[137] Cohn JS Tremblay M Batal R Jacques H Rodriguez C Steiner G. Increased apoC-III production is a characteristic feature of patients with hypertriglyceridemia. Atherosclerosis. (2004) 177:137–45. 10.1016/j.atherosclerosis.2004.06.011 15488876

[138] Marçais C Bernard S Merlin M Ulhmann M Mestre B Rochet-Mingret L. Severe hypertriglyceridaemia in type II diabetes: involvement of apoC-III Sst-I polymorphism, LPL mutations and apo E3 deficiency. Diabetologia. (2000) 43:1346–52. 10.1007/s001250051537 11126401

[139] Gaudet D Brisson D Tremblay K Alexander VJ Singleton W Hughes SG. Targeting APOC3 in the familial chylomicronemia syndrome. N Engl J Med. (2014) 371:2200–6. 10.1056/NEJMoa1400284 25470695

[140] Cohn JS Patterson BW Uffelman KD Davignon J Steiner G. Rate of production of plasma and very-low-density lipoprotein (VLDL) apolipoprotein C-III is strongly related to the concentration and level of production of VLDL triglyceride in male subjects with different body weights and levels of insulin sensitivity. J Clin Endocrinol Metab. (2004) 89:3949–55. 10.1210/jc.2003-032056 15292332

[141] Sundaram M Zhong S Khalil MB Links PH Zhao Y Iqbal J. Expression of apolipoprotein C-III in McA-RH7777 cells enhances VLDL assembly and secretion under lipid-rich conditions. J Lipid Res. (2010) 51:150–61. 10.1194/jlr.M900346-JLR200 19622837 PMC2789775

[142] Wang F Kohan AB Dong HH Yang Q Xu M Huesman S. Overexpression of apolipoprotein C-III decreases secretion of dietary triglyceride into lymph. Physiol Rep. (2014) 2:e00247. 10.1002/phy2.247 24760506 PMC4002232

[143] Ramms B Patel S Nora C Pessentheiner AR Chang MW Green CR. ApoC-III ASO promotes tissue LPL activity in the absence of apoE-mediated TRL clearance. J Lipid Res. (2019) 60:1379–95. 10.1194/jlr.M093740 31092690 PMC6672034

[144] Khetarpal SA Zeng X Millar JS Vitali C Somasundara AVH Zanoni P. A human APOC3 missense variant and monoclonal antibody accelerate apoC-III clearance and lower triglyceride-rich lipoprotein levels. Nat Med. (2017) 23:1086–94. 10.1038/nm.4390 28825717 PMC5669375

[145] Davies BSJ. Can targeting ANGPTL proteins improve glucose tolerance? Diabetologia. (2018) 61:1277–81. 10.1007/s00125-018-4604-4 29619530 PMC5940535

[146] Graham MJ Lee RG Brandt TA Tai L-J Fu W Peralta R. Cardiovascular and metabolic effects of *ANGPTL3* antisense oligonucleotides. N Engl J Med. (2017) 377:222–32. 10.1056/NEJMoa1701329 28538111

[147] Cushing EM Chi X Sylvers KL Shetty SK Potthoff MJ Davies BSJ. Angiopoietin-like 4 directs uptake of dietary fat away from adipose during fasting. Mol Metab. (2017) 6:809–18. 10.1016/j.molmet.2017.06.007 28752045 PMC5518724

[148] Mattijssen F Alex S Swarts HJ Groen AK Schothorst van EM Kersten S. Angptl4 serves as an endogenous inhibitor of intestinal lipid digestion. Mol Metab. (2014) 3:135–44. 10.1016/j.molmet.2013.11.004 24634819 PMC3953698

[149] Myocardial infarction genetics and cardiogram exome consortia investigators Coding variation in *ANGPTL4, LPL*, and *SVEP1* and the risk of coronary disease. N Engl J Med. (2016) 374:1134–44. 10.1056/NEJMoa1507652 26934567 PMC4850838

[150] Dewey FE Gusarova V O'Dushlaine C Gottesman O Trejos J Hunt C. Inactivating variants in *ANGPTL4* and risk of coronary artery disease. N Engl J Med. (2016) 374:1123–33. 10.1056/NEJMoa1510926 26933753 PMC4900689

[151] Zhang R. Lipasin, a novel nutritionally-regulated liver-enriched factor that regulates serum triglyceride levels. Biochem Biophys Res Commun. (2012) 424:786–92. 10.1016/j.bbrc.2012.07.038 22809513

[152] Dang F Wu R Wang P Wu Y Azam MdS Xu Q. Fasting and feeding signals control the oscillatory expression of ANGPTL8 to modulate lipid metabolism. Sci Rep. (2016) 6:36926. 10.1038/srep36926 27845381 PMC5109406

